# RE: ChatGPT can yield valuable responses in the context of orthopaedic trauma surgery

**DOI:** 10.1002/jeo2.70745

**Published:** 2026-05-07

**Authors:** Hinpetch Daungsupawong, Viroj Wiwanitkit

**Affiliations:** ^1^ Private Academic Consultan Phonhong Laos; ^2^ Medical College Saveetha Institute of Medical and Technical Sciences Saveetha University India Chennai India

Dear Editor,

‘ChatGPT can yield valuable responses in the context of orthopaedic trauma surgery [1]’ is an interesting article. By asking ChatGPT a series of questions that are frequently asked in the field of orthopaedic trauma surgery, the researchers investigated the potential application of the tool in this profession. Orthopaedic trauma specialists then assessed the ChatGPT responses for correctness, completeness, and adaptability for each of the three target groups. According to the results, ChatGPT functioned rather well; responses designed for non‐orthopaedic medical professionals and expert orthopaedic surgeons received higher scores for correctness, completeness, and adaptability than responses intended for patients.

A study finding highlights ChatGPT's inferiority in producing patient responses when compared to non‐orthopaedic physicians and skilled orthopaedic surgeons. This implies that in order to maximize ChatGPT's usefulness in this situation, more improvement and personalization of the responses tailored especially for patient education and communication may be required. Furthermore, no consideration was given to any restrictions or hurdles that can occur while utilizing ChatGPT in actual orthopaedic trauma surgery settings, such as patient‐specific quirks or customized treatment plans.

We would like to know if the study's authors have thought about adding feedback systems or continuous learning techniques to improve ChatGPT's functionality and adaptability over time. To make sure that ChatGPT's responses are accurate, current, and catered to the unique requirements of various target groups, this may entail feeding the model with more data on orthopaedic trauma surgery or integrating real‐time feedback from orthopaedic surgeons.

Further studies could look into how ChatGPT might be incorporated into clinical practice Complete Manuscript (Anonymized) 2 workflows to evaluate its effects on patient outcomes, surgeon productivity, and general care quality. Future study in this field may examine the viability of utilizing ChatGPT in telemedicine platforms, virtual consultation settings, or patient education tools to assist orthopaedic trauma surgeons in providing individualized, high‐quality care from a distance. Further research should focus on the moral and legal ramifications of deploying AI‐powered language models like ChatGPT in healthcare settings, especially with regard to patient privacy, data security, and liability. All things considered, the study's conclusions set the stage for further investigation into how best to use ChatGPT in orthopaedic trauma surgery as well as how to improve patient‐surgeon communication and decision‐making.

## ACKNOWLEDGEMENTS

The authors have no funding to report.

## CONFLICT OF INTEREST STATEMENT

The authors declare no conflicts of interest.
